# A Modified Technique of Percutaneous Fixation of Displaced Pediatric Lateral Humeral Condyle Elbow Fractures (aka the Martini Technique): A Brief Case Series

**DOI:** 10.5435/JAAOSGlobal-D-21-00293

**Published:** 2023-08-18

**Authors:** Thomas W. Hodo, Patrik Suwak, Theresa Lago, Tolulope Ramos, William Accousti

**Affiliations:** From the Department of Orthopedic Surgery, Tulane University School of Medicine, New Orleans, LA (Dr. Hodo and Dr. Ramos); and the Department of Orthopedic Surgery, Louisiana State University School of Medicine, New Orleans, LA (Dr. Suwak, Ms. Lago, and Dr. Accousti).

## Abstract

Displaced lateral humeral condyle (LHC) fractures have routinely been treated with open reduction, which has known postoperative complications. Recent reports show that closed reduction and percutaneous pinning (CRPP) is a valid treatment. Five pediatric patients with displaced LHC fractures were included in a retrospective case series. Closed reductions (CRs) were facilitated by Kirschner wire placement into the capitellum through a toothed drill guide. The Kirschner wire and drill guide were used like a joystick to manipulate the fragment and maintain reduction for placement of additional Kirschner wires. Patient records were used to determine the number of patients diagnosed with LHC fractures between 2011 to 2022 among six pediatric orthopaedic surgeons at one institution along with the treatment and associated complications. Satisfactory reduction of displaced LHC fractures was achieved with CRPP in all patients with no complications using the “martini” modification. Of 26 LHC fractures, 16 were treated with CRPP/CRPS and 10 with open reduction and percutaneous pinning/open reduction and internal fixation, with four converted from CR to OR. Complications included one superficial infection in the CR group and four stiff elbows and one nonunion in the OR group option for LHC fractures. CRPP is an effective treatment option with a decreased risk of complications. Our modified reduction technique may help improve the success and results of closed treatment of LHC fractures.

Fractures of the lateral humeral condyle (LHC) are common injuries that make up 10% to 20% of all pediatric elbow fractures, which is second only to supracondylar-type fractures.^1,2^ The mechanism of injury typically involves a fall with a varus force applied to the extended elbow causing an avulsion of the LHC fragment by the lateral collateral ligament complex and forearm extensor muscles.^3^ Impaction of the radial head into the lateral condyle has also been proposed as a mode of injury.

Several classification systems are commonly used to describe LHC fractures. The Milch^4^ classification defines the injury based on the anatomic relation of the fracture line to the trochlear groove. By contrast, the Jakob classification describes the fracture based on the degree of displacement and rotation, which helps guide treatment and correlates the clinical outcome.^3,5^ Weiss et al^6^ expand on the Jakob classification by including articular surface disruption visualized by an arthrogram to more accurately guide the treatment regimen. Finally, Song et al^7^ described a classification that expands on and more accurately describes unstable fracture patterns while offering a treatment algorithm.

Surgical options for the management of this injury include the standard open reduction and internal fixation (ORIF) or open reduction and percutaneous pinning (ORPP) and closed reduction and percutaneous pinning (CRPP) or closed reduction percutaneous screw fixation (CRPS). Common complications with either technique must be considered when selecting preferred management. Associated risks of open reduction (OR) include osteonecrosis, physeal arrest, arthrofibrosis, nonunion, and infection. By contrast, CRPP has been associated with a risk of malreduction. Malreduction may lead to not only malunion but also nonunion, physeal arrest with late valgus deformity, and spurring.^8^ Historically, most displaced lateral condyle fractures with rotation or articular surface disruption have been treated by open exposure because of the difficulty in reducing the fracture and the perceived need to visualize the articular surface. However, recent studies by Song et al and Weiss et al have shown that CRPP is a viable option with comparable outcomes.^6,7,9^

The purpose of this study was to describe a reproducible and simple modification of a previously described technique to more effectively achieve closed reduction (CR) and percutaneous fixation of significantly displaced lateral condyle fractures. Also, this study will compare closed and open techniques including the associated complications of each.

## Methods

All patients directly included in this study are pediatric patients (age 2 to 13 years) who received care at a single pediatric tertiary care facility (Children's Hospital of New Orleans) between 2011 and 2022. Institutional review board evaluation cleared the requirement of consent based on the designation as a retrospective analysis. The case series by our describing author includes five patients who sustained LHC fractures with notable displacement (>2 mm) and rotational malalignment: for example, Jakob stage 3/Song stage 4 or 5 unstable fractures. All other lateral condyle fractures not treated with this method were excluded. Each of the patients was treated by a single surgeon (W.A.). Three of the five patients were followed for at least 6 months after CRPP and up to 1 year.

The SlicerDicer feature of the Epic electronic medical record system was used as a database to further investigate the treatment of displaced lateral condyle fractures among six pediatric orthopaedic surgeons at Children's Hospital of New Orleans between 2011 and 2022. The set parameters of the patient demographics included all children up to the age of 16 years with a diagnosis of a displaced fracture of the lateral condyle of the humerus and initial encounter for closed fracture (International Classification of Diseases, 10th Revision, Clinical Modification [ICD‐10‐CM: S42.452 and S42.453]). Patients with these diagnoses were then queried for the procedures performed: ORIF and CRPP to determine how these fractures were treated. The database was also queried for postoperative complications associated with CRPP and ORIF, such as osteonecrosis of the humerus (*ICD-10-CM*: M87.9 and M87.22), physeal arrest of the distal humerus (*ICD-10-CM*: M89.12), nonunion (*ICD-10-CM*: S42.451K; S42.452K; and S42.453K), infection (*ICD-10-CM*: T81.40 and T81.41), and elbow deformity (*ICD-10-CM*: M21.02 and M21.12). Each patient's chart was further reviewed for the patient demographics, fracture classification, details of the procedure, follow-up, and complications.

The patient was placed supine on a radiolucent bed such as a flat-top table, with the fluoroscopic imaging device entering from the contralateral side of the injured extremity. No arm board was used. Draping in a typical sterile fashion should be performed in a manner that allows free movement and access to the entire arm.

**Figure 1:**
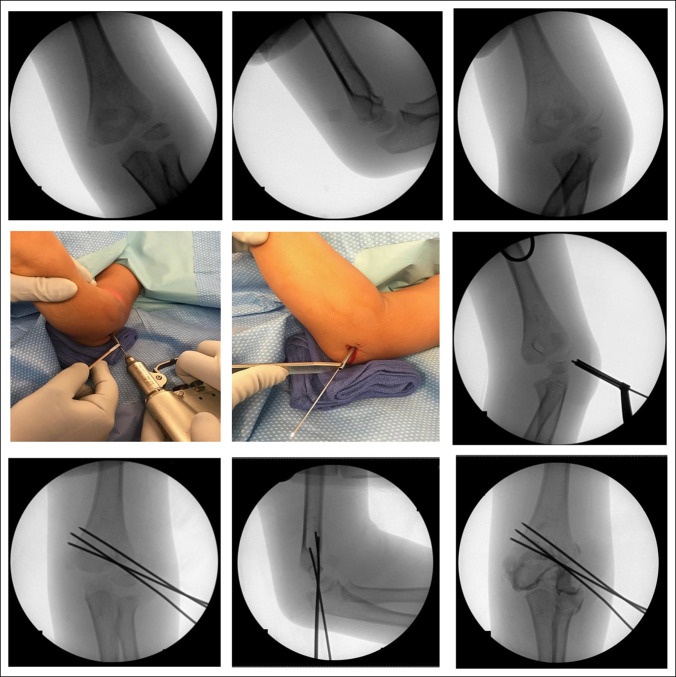
Fluoroscopy showing martini modification: **A**–**C,** AP, lateral, and oblique radiographs showing displaced and rotated lateral condyle fractures. **D,** 0.062-inch Kirschner wire placed into the lateral condyle fragment with a toothed drill over the wire for manipulation and stabilization. **E** and **F,** AP and lateral radiographs showing bicortical fixation with three Kirschner wires. **G,** AP arthrogram confirming acceptable reduction of the lateral condyle.

CR maneuvers should be attempted initially if notable displacement or rotation is present by applying traction with the elbow in extension using a slight varus force and direct manipulation of the displaced fragment anteromedially with the thumb (Figure 1A). If unsuccessful, a small incision (3.5 mm) is made over the lateral aspect of the elbow at the site of the displaced lateral condyle fragment. A smooth 0.062 Kirschner wire is then placed into the fractured fragment and capitellum in a position that will potentially allow its advancement into the proximal fragment, although that is not mandatory. Because this often looks like an olive being speared by a toothpick, it has been coined as the “martini technique” by the lead surgeon. A standard toothed drill sleeve (as found in many small fragment fixation sets) is then slid over the Kirschner wire and through the skin incision to engage the bone/cartilage surface to allow controlled “joysticking” of the fragment. If notable rotation is present, it is corrected initially by using the toothed drill sleeve and Kirschner wire to derotate the fragment. Then, with a combination of “joysticking” of the fragment, application of anteromedial pressure with the contralateral hand, and flexion/rotation maneuvers of the elbow by an assistant, the fragment can be reduced into an anatomic or near-anatomic position (Figure 1B). Two or three additional smooth 0.062 Kirschner wires should be placed percutaneously through the skin into the reduced fragment in a standard manner. These Kirschner wires should be passed through the far cortex to gain bicortical fixation, preferably in a diverging alignment, to provide stable fixation (Figure 1, C, D). This construct is typically used on small children or patients below age 7. 4.0mm or 4.5 mm cannulated screws can be used in larger or older (>7 years) children.

After confirmation of reduction with final radiographs (an arthrogram is not considered mandatory but can be performed at the surgeon's discretion), a long arm cast should be applied with the elbow in near 90° of flexion and bivalved, as deemed necessary based on swelling. Cast and pins are removed in the clinic at 3 to 4 weeks (if radiographically healed) with allowance of free active range of motion but limited weight bearing until 5 to 6 weeks. Follow-up should be conducted for at least 6 months to monitor for potential complications, such as physeal arrest, malunion, or nonunion.

## Results

In this series of five patients treated by the describing author with Jakob stage 3/Song stage 4 or 5 lateral condyle fractures with notable displacement and rotation, each was successfully reduced using the above-described “martini technique” with minimal to no residual displacement. The age of the patients treated with CRPP ranged from 3 to 6 years, with an average age of 5 years.

No other concomitant injuries were present in any of the patients. All fractures were reduced to <2 mm of displacement with no notable coronal or sagittal translation remaining. No loss of reduction occurred after CRPP in any of the patients. Notable healing was present in all fractures at 3 to 4 weeks of follow-up, mainly over the lateral cortex. Complete radiographic union occurred in all patients by 3 to 4 months.

**Figure 2:**
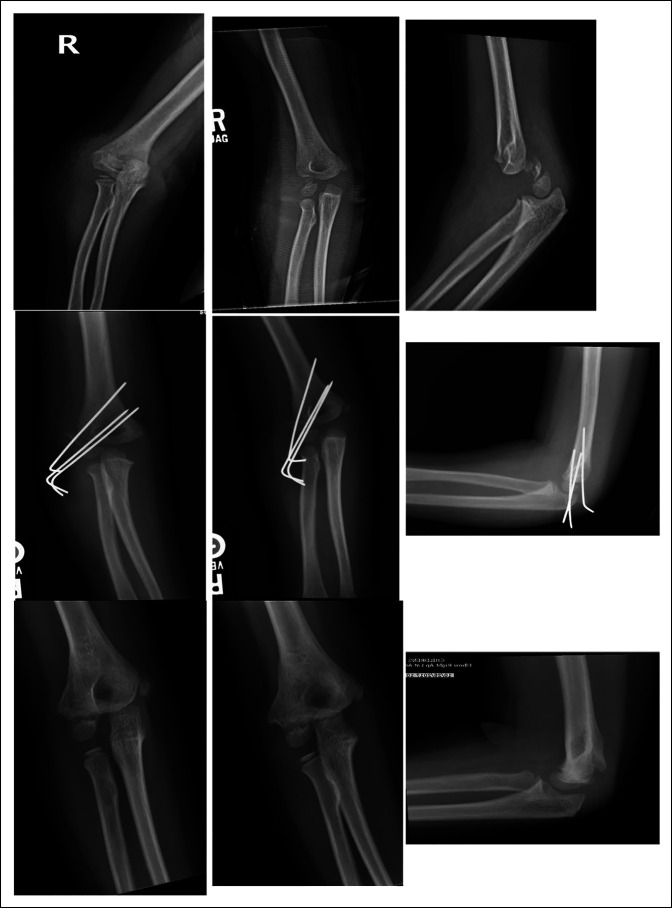
Three-year-old girl: **A**–**C,** AP, oblique, and lateral radiographs of initial injury. **D** and **E,** Three weeks post-op. **F**–**H,** Eleven months post-op with osteophyte formation posterior and laterally.

**Figure 3:**
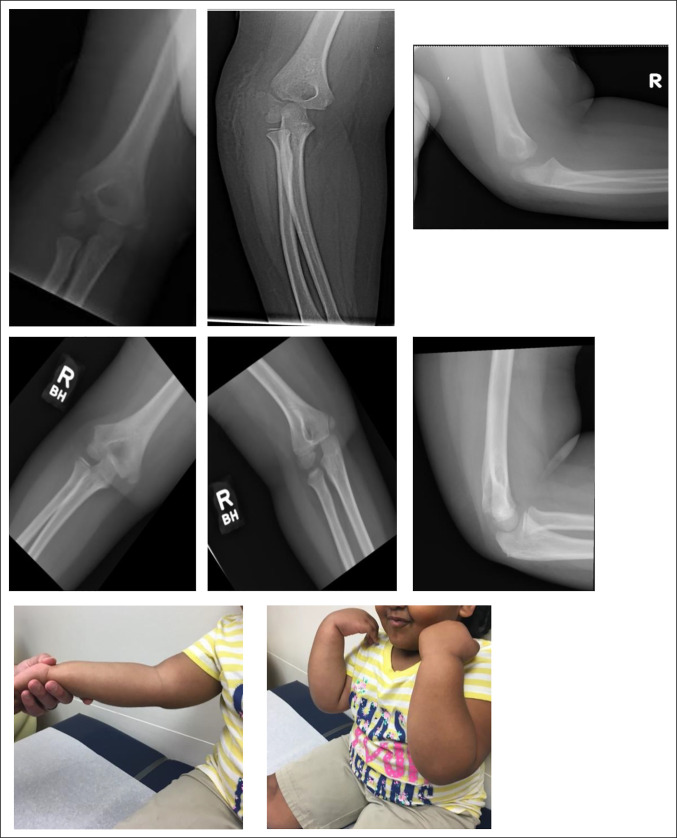
Five-year-old girl: **A**–**C,** AP, oblique, and lateral radiographs of initial injury. **D**–**F,** Nine months post-op. **G** and **H,** Full range of motion after CRPP.

No major complications such as infection, nonunion, malunion, or growth arrest were present in the series. Minor complications included one patient who developed radiographically visible posterior and lateral bony growth, although no gross clinical deformity or limitations to range of motion were present on examination (Figure 2, E,F). Another patient had a loss of 10° of active extension of the elbow at 3 months that subsequently resolved (Figure 3).

**Figure 4:**
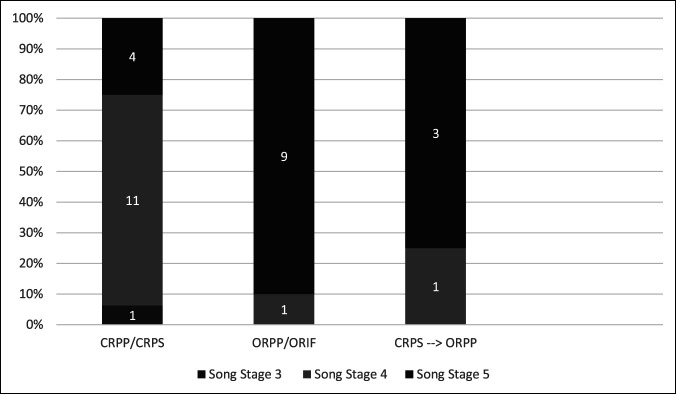
Bar graph showing closed reduction versus OR of displaced lateral condyle fractures. OR = open reduction.

To compare overall outcomes of CR versus OR techniques in unstable lateral condyle fractures, a retrospective analysis using electronic medical record data was performed. We identified 26 patients with displaced lateral condyle fracture (Song stage 3 or greater) treated with CRPP/CRPS or ORPP/ORIF by six pediatric orthopaedic surgeons at our institution, who used various methods of fixation. Their ages ranged from 2 to 13 years, with a mean age of 6 years. The LHC fractures included Song stages 3 to 5, with 1 stage 3, 12 stage 4, and 13 stage 5 fractures. The 1 stage 3 fracture and 11 of 12 stage 4 fractures were treated with CR, with 11 receiving percutaneous pin fixation and 1 receiving percutaneous screw fixation. Also, four stage 5 fractures were treated with CRPP, three of which were by the senior author and the other by another senior pediatric orthopaedic surgeon who used a similar method using a Kirschner wire to joystick the fracture fragment along with a Frazier suction tip for better control and compression. A total of one stage 4 and nine stage 5 fractures were treated with OR, with one fracture receiving screw fixation whereas all others were treated with percutaneous pin fixation. One stage 4 and three stage 5 fractures began with an attempt of CR but were converted to ORPP after inability to obtain a satisfactory reduction (Figure 4).

**Figure 5:**
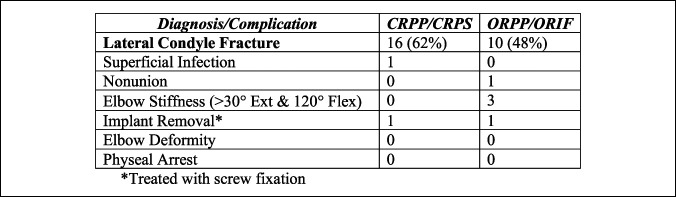
Treatment and complications of displaced lateral condyle fractures.

The average follow-up for all patients was 4 months, with ORPP/ORIF having a slightly longer follow-up time of 4.6 months compared with 3.9 months for CRPP/CRPS. There was one minor superficial pin-site infection from one patient treated with CRPP, which was treated with silver nitrate. There were three patients who had persistent elbow stiffness at 3 months, all of whom sustained stage 5 fractures and underwent ORPP or ORIF. One patient with stage 5 fracture developed a nonunion after ORPP and required a revision procedure with bone grafting and screw fixation (Figure 5). Two patients required implant removal: One was due to painful implant after CRPS, and the other was a planned removal after ORIF. None of the patients developed elbow deformity or physeal arrest during the observed follow-up time.

## Discussion

Historically, significantly displaced and rotated LHC fractures (Jakob stage 3/Song stage 4 or 5) have been treated with OR. However, OR has been associated with an increased risk of postoperative complications such as osteonecrosis, early physeal arrest, infection, and stiffness.

Pennock et al^8^ showed that unstable fractures displaced >2 mm treated with ORIF had an overall 25% complication rate compared with 13% when treated with CRPP. Major complications of ORIF included osteonecrosis with residual fishtail deformity, deep infection with early physeal arrest, and refracture. These major complications have not been described with CRPP.^8^ Minor complications such as stiffness and pin-site infections were also more common in the ORIF group.^8^ Thomas et al^10^ also showed that 40% (25/63) of patients developed overgrowth of the lateral condyle with ORIF leading to pseudocubitus varus or relative cubitus varus (13%), with one patient developing a radial nerve palsy and one developing a nonunion.

Recent studies have shown that unstable and displaced fractures can be treated with CRPP, potentially decreasing the risks associated with OR. Song et al showed a total success rate of 73% with CRPP of unstable/displaced lateral condyle fractures by a single surgeon. Unstable fractures in that study were described by a new classification by Song et al to denote stable and unstable fractures more accurately: Stage 3 fractures: <2 mm displacement with wide fracture propagation, stage 4: >2 mm displacement, and stage 5: >2 mm displacement with rotation, defined the unstable fractures. Reduction of all unstable fractures was attempted with 76% (13/17) of stage 3 fractures being reduced to <1 mm of residual displacement, whereas 75% (30/40) of stage 4 and 50% (3/6) of stage 5 fractures were reduced to <2 mm of residual displacement. CRPP was converted to ORIF for the remaining stage 4 (10) and stage 5 (3) fractures.^9^ No major complications were encountered. Minor complications included osteophyte formation (11) and mild capitellum hypertrophy (4). The cohort resulted in a 96% excellent clinical outcome according to the assessment by Hardacre et al.^9,11^

Song et al further showed CRPP of unstable lateral condyle fractures (Jakob stage 3/Song stage 4 or 5) to be a reproducible procedure by showing a 75% (18/24) success rate of acceptable reduction among three surgeons at different hospitals with a 94.4% (17/18) excellent clinical outcome.^9,11^ Three lateral condyle fractures were treated with ORIF early in the study likely because of the learning period needed to achieve satisfactory CR, which is a valid limitation and concern for any pediatric orthopaedic surgeon with limited experience in this approach. Our retrospective analysis of pediatric displaced lateral condyle fractures over the past 10 years at Children's Hospital of New Orleans showed CRPP to be the overall preferred method of fixation of all displaced lateral condyle fractures (Jakob stage 3/Song stage 4 to 5) with 62% of fractures treated with CRPP and 38% with ORIF. However, in Song stage 5 fractures with displacement and rotation, ORPP/ORIF is still preferred with only 7 of 13 attempted closed and 4 of 13 successfully treated closed. A total of 3 of the successful Song stage 5 fractures were treated by the senior author and the other fracture treated by another senior surgeon who used a similar method with a Frazier suction tip instead of a drill guide. These fractures were more likely to be treated closed by surgeons (>10 years after fellowship training), with 6 of 7 fractures attempted and 4 of 7 fractures successfully treated closed. Less experienced surgeons (<10 years) were more likely to perform OR, with only 1 of 6 fractures attempted closed before converting to open, and none were successfully treated closed. Although the fracture pattern and displacement have a likely role, our observation aligns with the previous literature that increasing experience allows for a higher success rate of closed treatment of Song stage 4 to 5/Jakob stage 3 fractures.^9^ Our retrospective analysis further proves CR to be advantageous with a lower likelihood of complications such as elbow stiffness and nonunion.

Song et al originally described the technique of CRPP of lateral condyle fractures. We provide a modification to Song's technique: the “martini technique” (as described by senior author W.A.) that can further assist orthopaedic surgeons in obtaining acceptable reductions even when notable initial displacement is present. For fractures that prove to be irreducible with CR and the “martini technique,” conversion to OR is indicated.

The “martini technique” uses a readily available drill sleeve (small fragment set) and supplements the original method by Song et al by allowing more controlled joysticking of the displaced lateral condylar fragment. Once the fragment is reduced, the drill guide also facilitates better compression across the fracture while additional smooth Kirschner wires (or screws) are inserted for fracture site fixation. Supplementary elbow flexion and supination/pronation maneuvers can be applied during reduction using the Kirschner wire and drill sleeve to assist in imparting added reduction forces on the lateral condyle fragment.

In conclusion, CRPP with the use of the described toothed drill sleeve and Kirschner wire (martini technique) allows for optimized reduction of displaced lateral condyle fragments that would otherwise necessitate formal OR. We agree with recent publications on the efficacy of CRPP for this fracture type and think that the described technique will lead to even higher rates of successful CR and subsequent improved outcomes by avoiding the well-described risks associated with OR.

This study is a limited case series of a single surgeon and retrospective analysis of six pediatric orthopaedic surgeons from one institution that has limitations to follow-up, which may not allow discovery of late complications such as physeal arrest and valgus deformity of the elbow.
